# Supplementary material for: Characterization of Sex-Based Differences in Gut Microbiota That Correlate with Suppression of Lupus in Female BWF1 Mice
Source: Microorganisms. 2025 Apr 29;13(5):1023. doi: 10.3390/microorganisms13051023 (PMC12113952; doi:10.3390/microorganisms13051023)
Supplement: Supplementary file 1 [file microorganisms-13-01023-s001.zip › microorganisms-3490782-supplementary.pdf]

microorganisms

MDPI

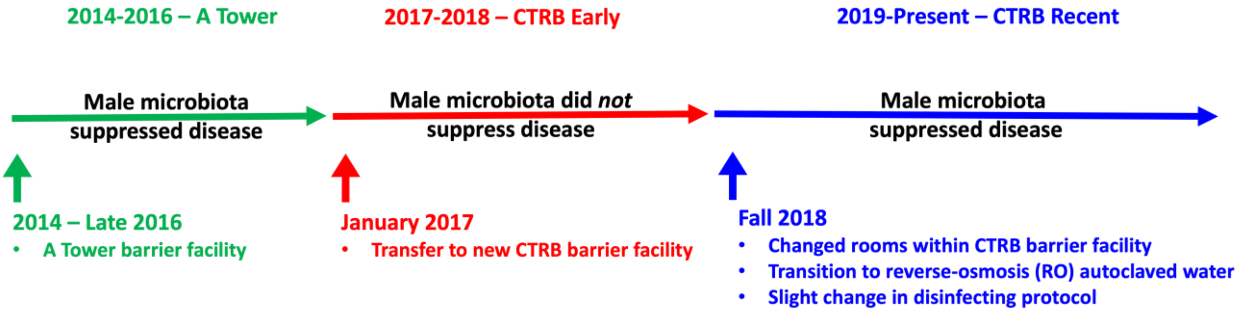


**Supplemental Figure S1.** Timeline of animal facility and disease suppression phenotype changes. Mice were housed in a specific pathogen-free animal facility in the A Tower building from 2014 to late 2016. During this period (referred to as 2014-2016 - A Tower), male microbiota transplants suppressed kidney disease in female BWF1 mice. Mouse colony was moved to another specific pathogen-free animal facility in the Clinical and Translational Research Building (CTRB) on the University of Louisville Health Sciences Campus in January, 2017. During this period between early 2017 and late 2018 (referred to as 2017-2018 - CTRB Early), male microbiota transplants lost the ability to suppress kidney disease in female BWF1 mice. Changes were made to the animal's environment and husbandry in the CTRB animal facility in the Fall of 2018. In early 2019 in the animal facility in the CTRB, male microbiota transplants regained the ability to suppress kidney disease in female BWF1 mice and that ability continues to the present (referred to as 2019-Present - CTRB Recent).

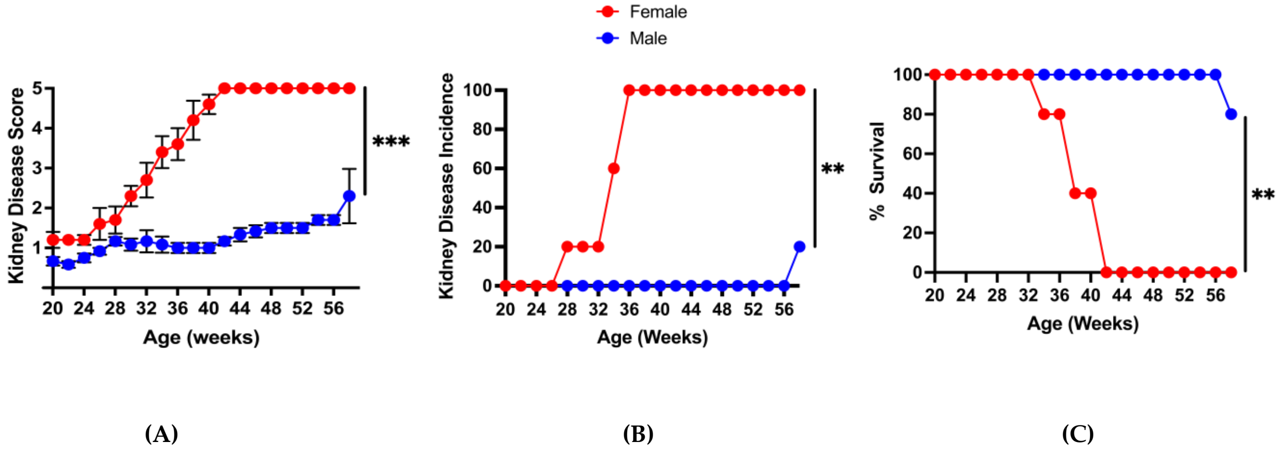


**Supplemental Figure S2.** Female, but not male, NZBxNZWF1 (BWF1) mice develop severe lupus nephritis spontaneously. Female and male BWF1 mice were monitored for kidney disease (glomerulonephritis) bi-weekly by measuring proteinuria in the urine. Proteinuria was scored on a scale of 1-5 with a score of 5 indicating death ($n$=5). (**A**) Kidney disease score (**B**) Incidence of kidney disease (% mice with a proteinuria score of ≥3 for two consecutive biweekly readings). (**C**) Survival curve. Kidney disease scores were compared by two-way ANOVA, and incidence and survival curves were compared using the Log rank (Mantel-Cox) test. *$p$<0.05, **$p$<0.01, ***$p$<0.001

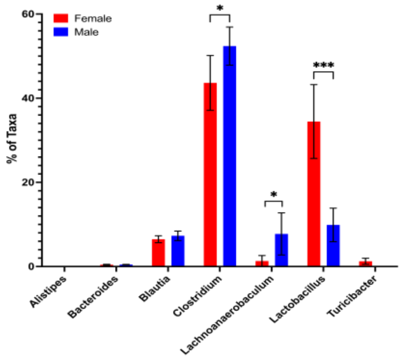


(A)

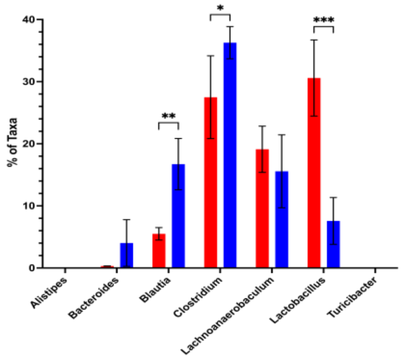


(B)

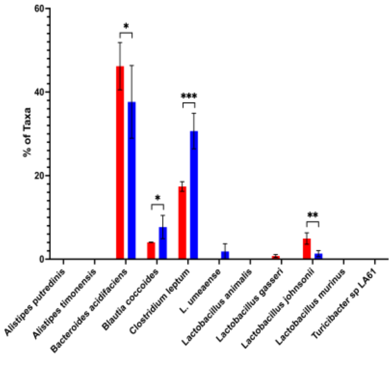


(C)

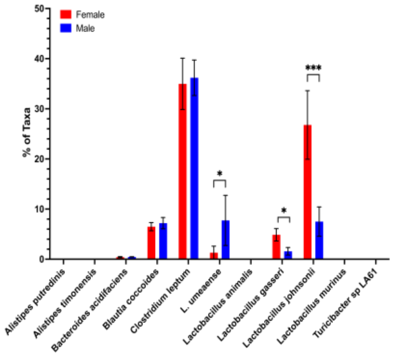


(D)

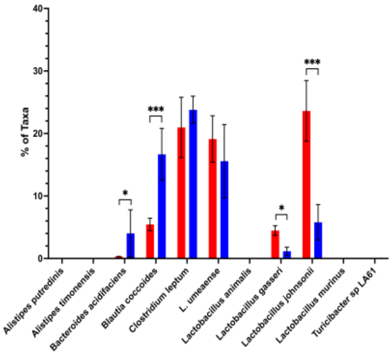


(E)

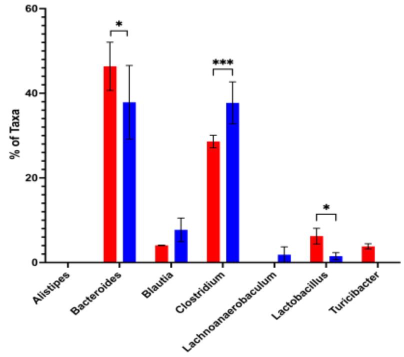


(F)

**Supplemental Figure S3.** *Bacteroides* is very low (with one exception) and abundance of *Clostridium* is very high in both females and males in most CTRB Early Experiments. Feces were collected from 16-week-old female and male BWF1 mice during the CTRB Early (2017-18) period and bacterial DNA was extracted. The 9 hypervariable regions of the 16S rRNA gene were sequenced and used to determine microbiota taxonomic composition. Abundances were compared with One-way ANOVAs. **(A)** Genus level microbiota from Experiment 2 ($n$=7). **(B)** Genus level microbiota from Experiment 3 ($n$=7). **(C)** Genus level microbiota from Experiment 4 ($n$=6). **(D)** Species level microbiota from Experiment 2 ($n$=7). **(E)** Species level microbiota from Experiment 3 ($n$=7). **(F)** Species level microbiota from Experiment 4 (n=6). *$p$<0.05, **$p$<0.01, ***$p$<0.001

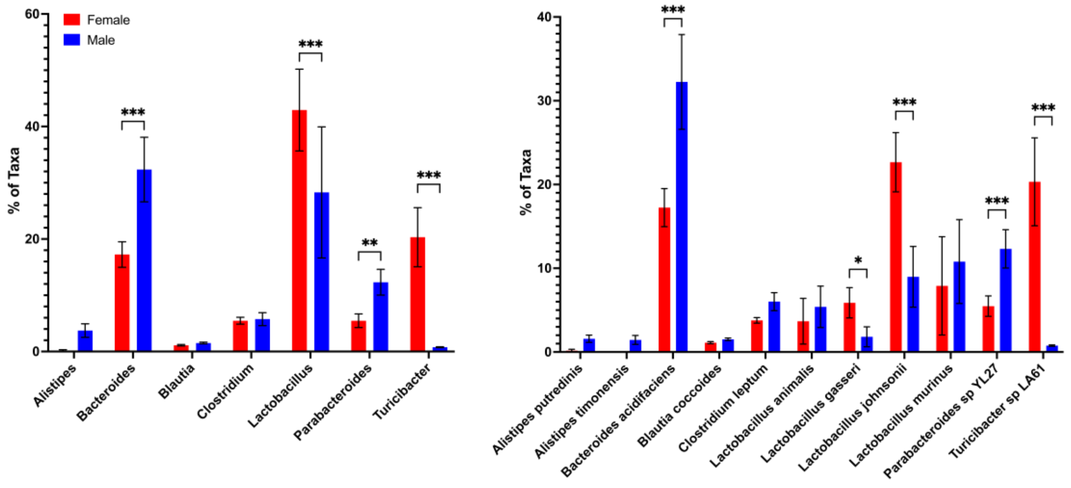


(A) (B)

**Supplemental Figure S4.** Abundance of *Bacteroides* was considerably higher in adult male than female BWF1 mice, and abundance of *Clostridium* levels was low in CTRB Recent Period. Feces were collected from 16-week-old female and male BWF1 mice during the CTRB Recent (2019-present) period and bacterial DNA was extracted. The 9 hypervariable regions of the 16S rRNA gene were sequenced and used to determine microbiota taxonomic composition. Abundances were compared with One-way ANOVAs. **(A)** Genus level microbiota from Experiment 2 ($n$=6). **(B)** Species level microbiota from Experiment 2 ($n$=6). *$p$<0.05, **$p<0.01$, ***$p$<0.001

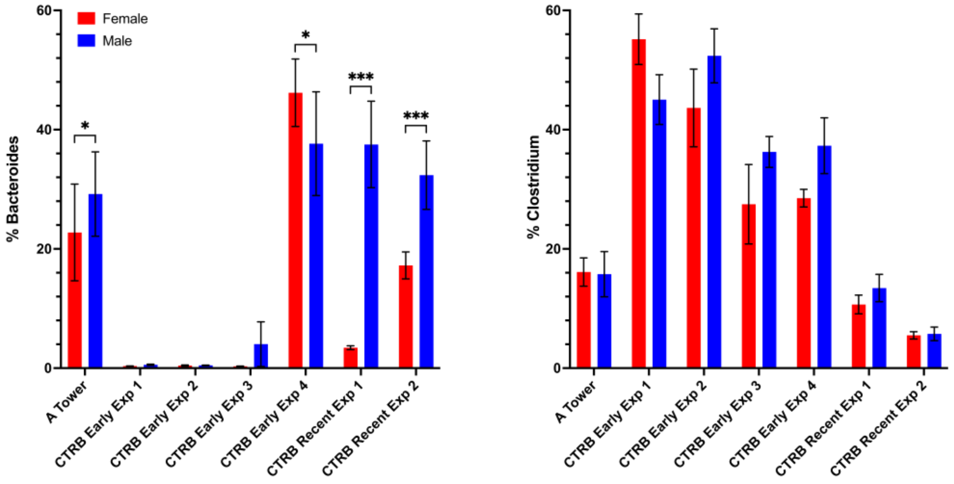


(A) (B)

**Supplemental Figure S5.** Comparison of *Bacteroides* and *Clostridium* abundance data in female and male BWF1 mice from experiments conducted during the A Tower, CTRB Early and CTRB Recent periods. Feces were collected from female and male BWF1 mice during the A Tower (2014-16), CTRB Early (2017-2018) and CTRB Recent (2019-present) periods and bacterial DNA was extracted. The 9 hypervariable regions of the 16S rRNA gene were sequenced and used to determine microbiota taxonomic composition. Abundances were compared with One-way ANOVAs. Data were compiled from experiments shown in **Figures 7, 8, 9** and **Supplemental Figures S3 and S4**. **(A)** Compilation of data for *Bacteroides* abundances in females and males; **(B)** Compilation of data for *Clostridium* abundances in females and males. *$p$<0.05, ***$p$<0.001

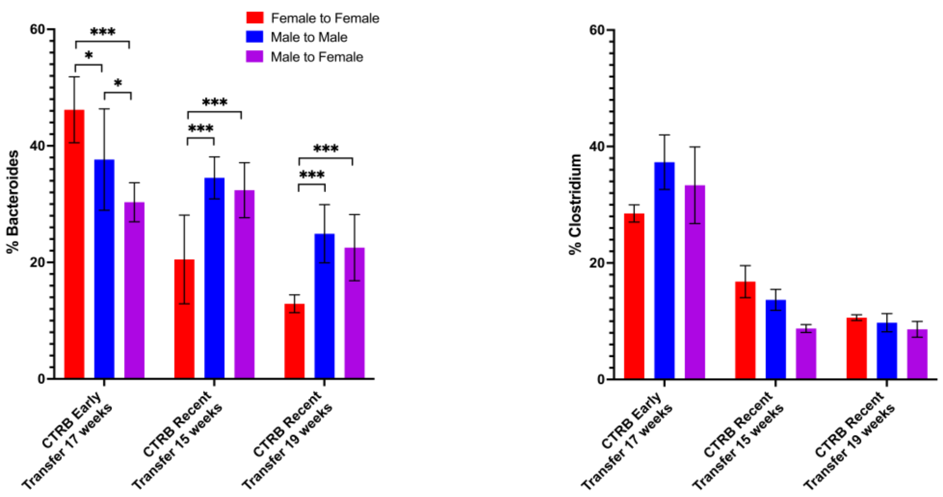


(A) (B)

**Supplemental Figure S6.** Comparison of *Bacteroides* and *Clostridium* abundance data in cecal transfer experiments conducted during the CTRB Early and CTRB Recent periods. During the CTRB Early (2017-2018) and CTRB Recent (2019-present) periods, female BWF1 mice were fed cecal contents (i.e., microbiota) from 16-week-old female (Female-to-Female) or male (Male-to-Female) BWF1 mice via gavage. Feces were collected from 16-week-old cecal transfer recipients (4 weeks after cecal transfer), and bacterial DNA was extracted. The 9 hypervariable regions of the 16S rRNA gene were sequenced and used to determine microbiota taxonomic composition. Abundances were compared with One-way ANOVAs. Data were compiled from experiments shown in **Figures 10** and **11**. **(A)** Compilation of data for *Bacteroides* abundances in cecal recipients; **(B)** Compilation of data for *Clostridium* abundances in cecal recipients. *$p$<0.05, ***$p$<0.001

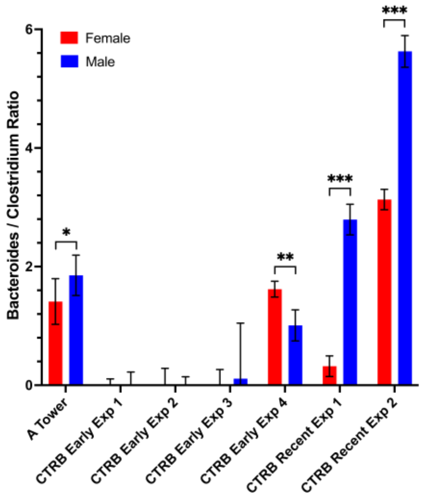


**Supplemental Figure S7.** Ratios between *Bacteroides* and *Clostridium* abundance in female vs male experiments. Fecal samples from females and males collected during the A Tower, CTRB Early (2017-2018) and CTRB Recent (2019-present) periods and bacterial DNA was extracted. The 9 hypervariable regions of the 16S rRNA gene were sequenced and used to determine microbiota taxonomic composition. The *Bacteroides/Clostridium* ratios were calculated from the data shown in **Figures 7, 8, 9 and Supplemental Figures S3 and S4** by dividing average *Bacteroides* abundance by average *Clostridium* abundance. *Bacteroides/Clostridium* ratios for the female to male comparisons and shown as *Bacteroides/Clostridium* ratios. *$p$<0.05, **$p$<0.01, ***$p$<0.001
